# Additive and heterozygous (dis)advantage GWAS models reveal candidate genes involved in the genotypic variation of maize hybrids to *Azospirillum brasilense*

**DOI:** 10.1371/journal.pone.0222788

**Published:** 2019-09-19

**Authors:** Miriam Suzane Vidotti, Danilo Hottis Lyra, Júlia Silva Morosini, Ítalo Stefanine Correia Granato, Maria Carolina Quecine, João Lúcio de Azevedo, Roberto Fritsche-Neto

**Affiliations:** 1 Department of Genetics, “Luiz de Queiroz” College of Agriculture, University of São Paulo, Piracicaba, São Paulo, Brazil; 2 Rothamsted Research, Harpenden, Hertfordshire, England, United Kingdom; 3 French National Institute for Agricultural Research, Montpellier, France; Osmania University, INDIA

## Abstract

Maize genotypes can show different responsiveness to inoculation with *Azospirillum brasilense* and an intriguing issue is which genes of the plant are involved in the recognition and growth promotion by these Plant Growth-Promoting Bacteria (PGPB). We conducted Genome-Wide Association Studies (GWAS) using additive and heterozygous (dis)advantage models to find candidate genes for root and shoot traits under nitrogen (N) stress and N stress plus *A*. *brasilense*. A total of 52,215 Single Nucleotide Polymorphism (SNP) markers were used for GWAS analyses. For the six root traits with significant inoculation effect, the GWAS analyses revealed 25 significant SNPs for the N stress plus *A*. *brasilense* treatment, in which only two were overlapped with the 22 found for N stress only. Most were found by the heterozygous (dis)advantage model and were more related to exclusive gene ontology terms. Interestingly, the candidate genes around the significant SNPs found for the maize–*A*. *brasilense* association were involved in different functions previously described for PGPB in plants (e.g. signaling pathways of the plant's defense system and phytohormone biosynthesis). Our findings are a benchmark in the understanding of the genetic variation among maize hybrids for the association with *A*. *brasilense* and reveal the potential for further enhancement of maize through this association.

## Introduction

Currently, major agro-systems are highly dependent on chemical fertilizers and pesticide inputs. One of the main strategies to develop sustainable agriculture in the face of natural resource scarcity and environmental impacts caused by the application of these products is the use of Plant Growth-Promoting Bacteria (PGPB) inoculants. These bacteria in association with plants may generate several benefits to the host, such as phytohormone biosynthesis, biological nitrogen fixation (BNF), and induction of resistance mechanisms. Consequently, there are positive effects on the enhancement of root traits, tolerance to abiotic stress, and defense against pathogens [1,2].

*Azospirillum brasilense* is a well-known PGPB marketed by several companies in South American countries (e.g. Brazil, Argentina, and Uruguay). It is used as a inoculant in some cereal crops such as maize and wheat [3]. Some studies have reported the influence of plant genotype on the degree of beneficial response to PGPB inoculation, including *A*. *brasilense* [4–6]. In this context, *Genome-wide Association Studies* (GWAS) is a powerful approach for the identification of genomic regions associated significantly with phenotypic trait variations and has been widely applied in the study of the genetic basis of plant–microbe interactions, including pathogens [7,8] arbuscular mycorrhizal fungi [9,10], and endogenous microbiomes [11]. As far as we know, only two GWAS studies were reported to PGPB. The first explored traits related to the BNF of *Rhizobium tropici* in a panel of 259 common beans [12]. The second evaluated shoot and root traits of 302 accessions of *Arabidopsis thaliana* inoculated with *Pseudomonas simiae* WCS417r [13]. However, GWAS studies related to genetic basis of cereals for the responsiveness to PGPB have not been reported so far, particularly for those with N-fixing ability.

Moreover, the growing of plants on unsterilized soil should be considered in studies concerning the relationship of plants with PGPB. The soil characteristics may influence this association, particularly due to the interaction of the inoculated strain with the soil microbiome. For instance, they might compete for resources and site, or show antagonist effects [14]. The understanding of the plants’ genetic basis related to PGPB and nitrogen (N) starvation is also crucial. It is known that changes in the diversity and the amount of the compounds released by the roots depend on the nutritional status, with consequences for the transcription of PGPB genes [15] and the composition of the plant-associated microbiome [16,17]. Furthermore, in tropical areas such as Africa and parts of South America, the soils are often N-limited and a significant proportion of maize production takes place under these conditions.

Another challenge is the heterosis (or hybrid vigor) of several maize traits [18–20]. Therefore, GWAS analyses should consider not only the additive marker effects but also the non-additive ones that might explain an important proportion of the variation in complex traits [21,22]. In this way, some authors speculate that the colonization of maize roots by beneficial microbes could be regulated by heterosis, due to hybrid plants supporting more numerous strains than their parental inbred lines [23,24]. In addition, studies of mechanisms underlying heterosis have shown changes, for example, in the expression patterns of hormone defense pathways and auxin biosynthesis [25], carbohydrate and nitrogen metabolism [26], besides increase of root and shoot biomass [27,28], which may also be related to plant responses to PGPBs [29–32]. However, this was not clearly elucidated. Thus, heterozygous (dis)advantage GWAS models [33,34] applied to the plant-related traits of the responsiveness to PGPB could provide additional information about the influence of heterosis concerning this association and help to identify candidate genes with heterotic performance under the inoculation conditions.

Knowledge about the genetic variation available and the genetic architecture of the traits involved in maize‒*A*. *brasilense* interaction is absent. However, this information can contribute to the understanding of its genetic base and how to apply it in plant breeding programs aimed at improving the germplasm for this association. Hence, we aimed with this study *(i)* to understand the genetic variation of maize in response to *A*. *brasilense* inoculation under low-N soil conditions, and *(ii)* to perform GWAS analyses using additive and heterozygous (dis)advantage models to identify Single Nucleotide Polymorphisms (SNPs) significantly associated with traits related to this association, the underlying candidate genes, and the importance of non-additive effects on the maize–*A*. *brasilense* association.

## Materials and methods

### Bacterial strain and inoculum

The bacterial strain *A*. *brasilense* Ab-V5 was selected from maize roots in Brazil and is registered by the Brazilian Ministry of Agriculture, Livestock and Food Supply (MAPA) for the inoculant production for maize, rice and wheat [3,35]. In addition, it is part of the Culture Collection of Diazotrophic and PGPB of Embrapa Soybean (Londrina, Paraná, Brazil). The bacterial inoculum of *A*. *brasilense* Ab-V5 was prepared in the Laboratory of Genetics of Microorganisms “Prof. João Lúcio de Azevedo” at ESALQ/USP, Piracicaba-SP, Brazil, and taken immediately to the experimental area. Bacterial inoculum was prepared by growing Ab-V5 in Dextrose Yeast Glucose Sucrose (DYGS) liquid medium [36] at 28°C with 150 rpm agitation. The inoculum concentration was adjusted to approximately 1 × 10^8^ UFC mL^–1^ and transferred with a pipette into plastic bags containing the maize seeds of each genotype individually. Sowing was done about 30 min after inoculation.

### Plant material and greenhouse experiments

The association panel was comprised of 118 single-cross hybrids from a diallel mating design between 19 tropical maize inbred lines with genetic diversity to nitrogen-use efficiency [37–39]. The plants were grown under semi-controlled conditions in a greenhouse located at the University of São Paulo, Brazil (22° 42' 39" S; 47° 38' 09" W, altitude 540 m), in two years: November–December 2016 and February–March 2017. A randomized complete block experimental design with three replications spatially arranged under two countertops was adopted in each season. Two main treatments were evaluated: N stress without bacterial inoculation and N stress plus *A*. *brasilense* inoculation. The decision to non-input N fertilizer was due to its reported negative effects on N fixation by diazotrophic bacteria [40,41]. In each plot, three seeds were sown at 3 cm depth in plastic pots of 3 L capacity containing unsterilized loam soil from an area not in agricultural use. Information about the soil chemical and physical characteristics is available in Vidotti et al. [42]. After germination, the seedlings were thinned singly. Only potassium chloride and single phosphate fertilizers were added to the soil according to the general crop demand. The average temperature was semi-controlled (20–33°C), and supplementation of luminosity was by fluorescent lamps to simulate a photoperiod of 12 h. The water supply was provided manually by pot, with the same amount applied to all of them and always maintaining a well-watered condition. During the conduction of the experiments, no insect or pathogen attack was detected, and pesticides were not used.

Approximately 35 days after the emergence, when most of the hybrids had reached the V7 stage (seven expanded leaves), plant height (PH, cm) was measured from the soil level to the insertion of the least expanded leaf. The shoot was harvested and dried in a forced-draft oven at 60°C for 72 h to determine the shoot dry mass (SDM, g). The soil particles in each root system were carefully removed with water and the individual storage was performed inside plastic pots in 25% ethanol solution for preservation. The root images acquired by an Epson LA2400 scanner (2400 dpi resolution) were analyzed by WinRHIZO^TM^ (Reagent Instruments Inc., Quebec, Canada). This software provided the measurements of root average diameter (RAD, mm), root volume (RV, cm^3^), and the total length of a series of root diameter classes. The length of fragments with a diameter class less than or equal to 0.5 mm were considered as the lateral root length (roots from the axial roots—LRL, cm), while that with diameter classes greater than 0.5 mm were considered as axial root length (comprising crow, seminal and primary roots—ARL, cm) [43]. We determined the root dry mass (RDM, g) after drying the roots under the same conditions used for SDM measurement. This trait was used to calculate the specific root length (SRL, cm g^–1^) and specific root surface area (SRSA, cm^2^ g^–1^) dividing the total root length and the surface area by the RDM, respectively. Furthermore, the root/shoot ratio (RSR, g g^–1^) was obtained by dividing the RDM by the SDM. In total, 10 traits were evaluated and approximately 1416 root systems analyzed.

### Phenotypic analyses

The analyses were conducted using Restricted Maximum Likelihood/Best Linear Unbiased Predictor (REML/BLUP) mixed models, by ASReml R package [44], considering the following model:

$$y=X_{E}\beta_{E}+X_{B}\beta_{B}+X_{C}\beta_{C}+X_{I}\beta_{I}+X_{EI}\beta_{EI}+Z_{G}u_{G}+Z_{GE}u_{GE}+Z_{GI}u_{GI}+Z_{GEI}u_{GEI}+\;\varepsilon$$

where ***y*** is the vector of phenotypic observations of the traits evaluated on maize hybrids; ***β***_***E***_ is the vector of fixed effects of year; ***β***_***B***_ is the vector of fixed effects of block within year; ***β***_***C***_ is the vector of fixed effects of countertop within block and year; ***β***_***I***_ is the vector of fixed effects of inoculation; ***β***_***EI***_ is the vector of fixed effects of inoculation × year interaction; ***u***_***G***_ is the vector of random effects of genotype, where $u_{G}\sim N\left(0,\;I\sigma_{G}^{2}\right)$; ***u***_***GE***_ is the vector of random effects of genotype × year interaction, where $u_{GE}\sim N\left(0,\;\sigma_{GE}^{2}\right)$; ***u***_***GI***_ is the vector of random effects of genotype × inoculation interaction, where $u_{GI}\sim N\left(0,\;\sigma_{GI}^{2}\right)$; ***u***_***GEI***_ is the vector of random effects of genotype × year × inoculation interaction, where $u_{GEI}\sim N\left(0,\;\sigma_{GEI}^{2}\right)$; and **ε** is the vector of errors, where $\varepsilon \sim N\left(0,\;\sigma_{\varepsilon }^{2}\right)$. ***X***_***E***_, ***X***_***B***_, ***X***_***C***_, ***X***_***I***_, ***X***_***EI***_, ***Z***_***G***_, ***Z***_***GE***_, ***Z***_***GI***_, and ***Z***_***GEI***_ are the respective incidence matrices related to each vector. The significance of fixed effects was tested using the Wald test implemented in the ASReml R package, while the significance of random effects was assessed by Likelihood Ratio Test (LTR) from the asremlPlus R package [45]. The variance components by treatment were estimated through reduced models disregarding the inoculation effect and its interaction with genotype. Broad-sense heritabilities were estimated as $H^{2}=\sigma_{G}^{2}/(\sigma_{G}^{2}+\sigma_{GE}^{2}/j+\sigma_{\varepsilon }^{2}/rj)$, where the $\sigma_{G}^{2}$ is the genetic variance; $\sigma_{GE}^{2}$ is the genotype-by-year variance; $\sigma_{\varepsilon }^{2}$ is the error variance; *j* and *r* are the number of years and replications in each experiment, respectively.

### Genotypic data

The Affymetrix^®^ Axiom^®^ Maize Genotyping Array [46] of 616,201 SNPs markers was used to genotype the parental inbred lines. Markers with call rate <95% and heterozygous loci on at least one individual were removed. Remaining missing data were imputed through the algorithms of Beagle 4.0 using the codeGeno function from the Synbreed R package [47]. The hybrid genotypes were obtained *in silico* from the genotypes of the corresponding parental inbred lines. After that, one more filter was applied to the matrix, eliminating SNPs with Minor Allele Frequency (MAF) ≤ 0.05. A final SNP set of 59,215 was obtained and used for the subsequent analyses.

### GWAS analyses

Marker-trait association analyses were performed for the traits with significant inoculation effect. For these traits, the adjusted means for each hybrid were calculated by treatment (inoculated and non-inoculated), separately, considering the following model:

$$y=X_{E}\beta_{E}+X_{B}\beta_{B}+X_{C}\beta_{C}+X_{G}\beta_{G}+X_{GE}\beta_{GE}+\varepsilon$$

where ***y*** is the vector of phenotypic observations of the traits evaluated on maize hybrids; ***β***_***E***_ is the vector of fixed effects of year; ***β***_***B***_ is the vector of fixed effects of block within year; ***β***_***C***_ is the vector of fixed effects of countertop within block and year; ***β***_***G***_ is the vector of fixed effects of the genotype; ***β***_***GE***_ is the vector of fixed effects of genotype × year interaction; and **ε** is the vector of errors, where $\varepsilon \sim N\left(0,\;\sigma_{\varepsilon }^{2}\right)$. ***X***_***E***_, ***X***_***B***_, ***X***_***C***_, ***X***_***G***_, and ***X***_***GE***_ are the respective incidence matrices for each vector. Density and box plots were used to compare the means between both treatments. In addition, the changes due to *A*. *brasilense* inoculation on the hybrid traits were calculated by Δ = *M*1−*M*2, where *M*1 is the adjusted mean under N stress plus *A*. *brasilense* and *M*2 is the adjusted mean under N stress.

Population structure was estimated by principal component analysis (PCA) using the genomic matrix through the SNPRelate R package [48]. The GWAS analyses were conducted by a Fixed and Random Model Circulating Probability Unification method thought the FarmCPU R package [49]. This statistical procedure considers the confounding effect between the testing marker and both kinship (K) and population structure (Q) as covariates to minimize the problem of false positive and false negative SNPs. The FarmCPU R package uses the FaST-LMM algorithm to calculate the K from selected pseudo-QTNs (Quantitative Trait Nucleotides) and not from the total SNP set, as the standard K. The threshold values were calculated by the p.threshold function of FarmCPU. This permutes the phenotypes to break any spurious relationship with the genotype. After obtaining a vector of the minimum p-values of each experiment, the 95% quantile value of the vector is recommended for p.threshold. Finally, quantile–quantile (Q–Q) plots were used to verify the fitness of the model, considering population structure and kinship as factors.

The additive and heterozygous (dis)advantage models were applied in GWAS analyses by using specific encodings for the SNP matrix. Concerning the additive SNP effect with two alleles (A_1_ and A_2_), the SNP matrix was coded by 0 (A_1_A_1_), 1(A_1_A_2_), and 2 (A_2_ A_2_), considering the A_2_ as the minor allele. In this context, the additive GWAS model assumes there is a linear change in the phenotype regarding the minor allele number of copies. On the other hand, in the heterozygous (dis)advantage GWAS model, the homozygous genotypes (A_1_A_1_ or A_2_A_2_) were assumed to have the same effect, while the heterozygous genotypes a different one, implying an increase or decrease of the effect on the trait. Therefore, the SNP matrix was coded by 0 (A_1_A_1_), 1 (A_1_A_2_), and 0 (A_2_A_2_) [33,34]. Box plots were then used to show the phenotype values by genotypes of the SNPs significantly associated with the traits.

The average linkage disequilibrium (LD) in the hybrid panel was investigated using the square allele frequency correlation coefficient *r*^2^ between all pairs of SNPs across the chromosomes using PLINK v.1.9 software [50]. The extension of LD decay was verified by plotting the *r*^2^ values against the physical distance of the SNPs. Moreover, the heterozygosity by hybrid and by SNP marker was estimated dividing the number of heterozygous loci by the total of SNP markers and maize genotypes, respectively.

### Identification of candidate genes

The candidate genes associated with the significant SNPs were obtained from the B73 genome reference (version 4) in the MaizeGDB genome browser (https://www.maizegdb.org/). Complementary information was collected from the U.S. National Center for Biotechnology Information (http://www.ncbi.nlm.nih.gov/) and the Universal Protein Resource (http://www.uniprot.org/). Venn diagrams were constructed to summarize the number of candidate genes identified using the VennDiagram R package [51]. In addition, the sequences of the candidate genes were categorized functionally by Gene Ontology (GO) terms [52], disregarding those with hypothetical function. The terms were obtained using the Blast2GO software with the default parameters specified by the program [53] and were previously simplified using the GO Slim feature.

## Results

### The phenotypic effect of *A*. *brasilense* inoculation on the maize hybrids

Significant phenotypic differences among the 118 maize hybrids were observed for all traits evaluated, except PH and SDM (S1 Table). Furthermore, the genotypic performance for RDM, RV, RAD, SRL, SRSA, and RSR were affected significantly by the inoculation with *A*. *brasilense*; thus, only these traits were considered for subsequent analyses. In general, higher broad-sense heritabilities were found under inoculated treatment than non-inoculated (S1B Fig).

**Fig 1:**
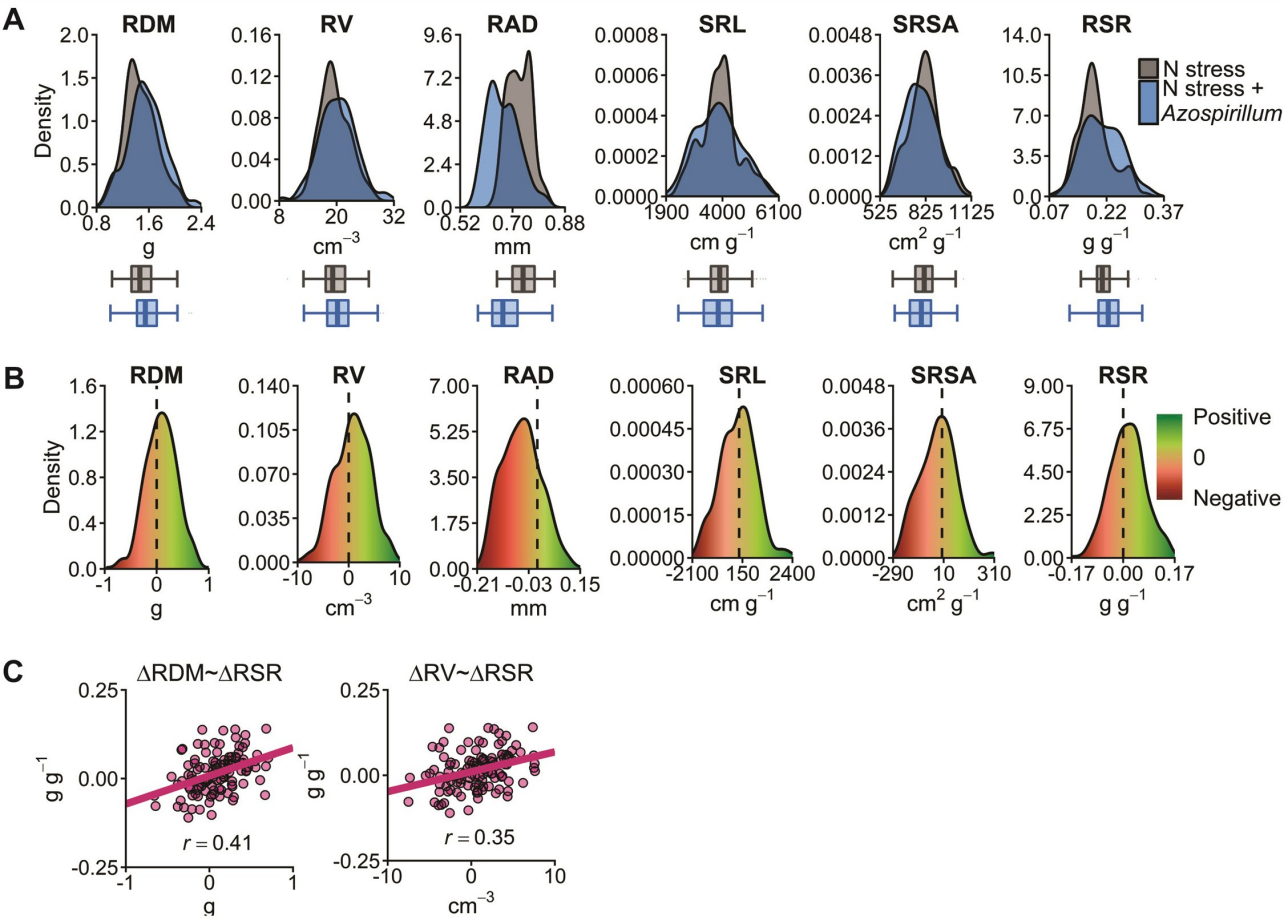
Density distributions and Pearson correlations of the phenotypic data. (A) Density distribution and box plot of the maize hybrids adjusted means under N stress and N stress plus *A*. *brasilense*. (B) Density distribution of the Δ (difference between adjusted means of inoculated and non-inoculated treatments). (C) Pearson correlations between Δ values.

Regarding the density distribution of the adjusted means for all traits, larger phenotypic variances were found in the inoculated condition compared to the non-inoculated (Fig 1A). Overall, the inoculation increased the RDM, RV, RAD, and RSR while the opposite was observed for SRL and SRSA. Concerning the change due to inoculation (Δ) for all traits, a distribution close to normal was observed (Fig 1B). In this sense, most of the evaluated hybrids showed low responsiveness to *A*. *brasilense*. Moreover, a considerable portion of the genotypes showed negative responsiveness to *A*. *brasilense*; that is, a worse performance than the non-inoculated. The correlations between the ΔRDM and ΔRV with ΔRSR were 0.41 and 0.35, respectively (Fig 1C).

### Population structure and LD decay

**Fig 2:**
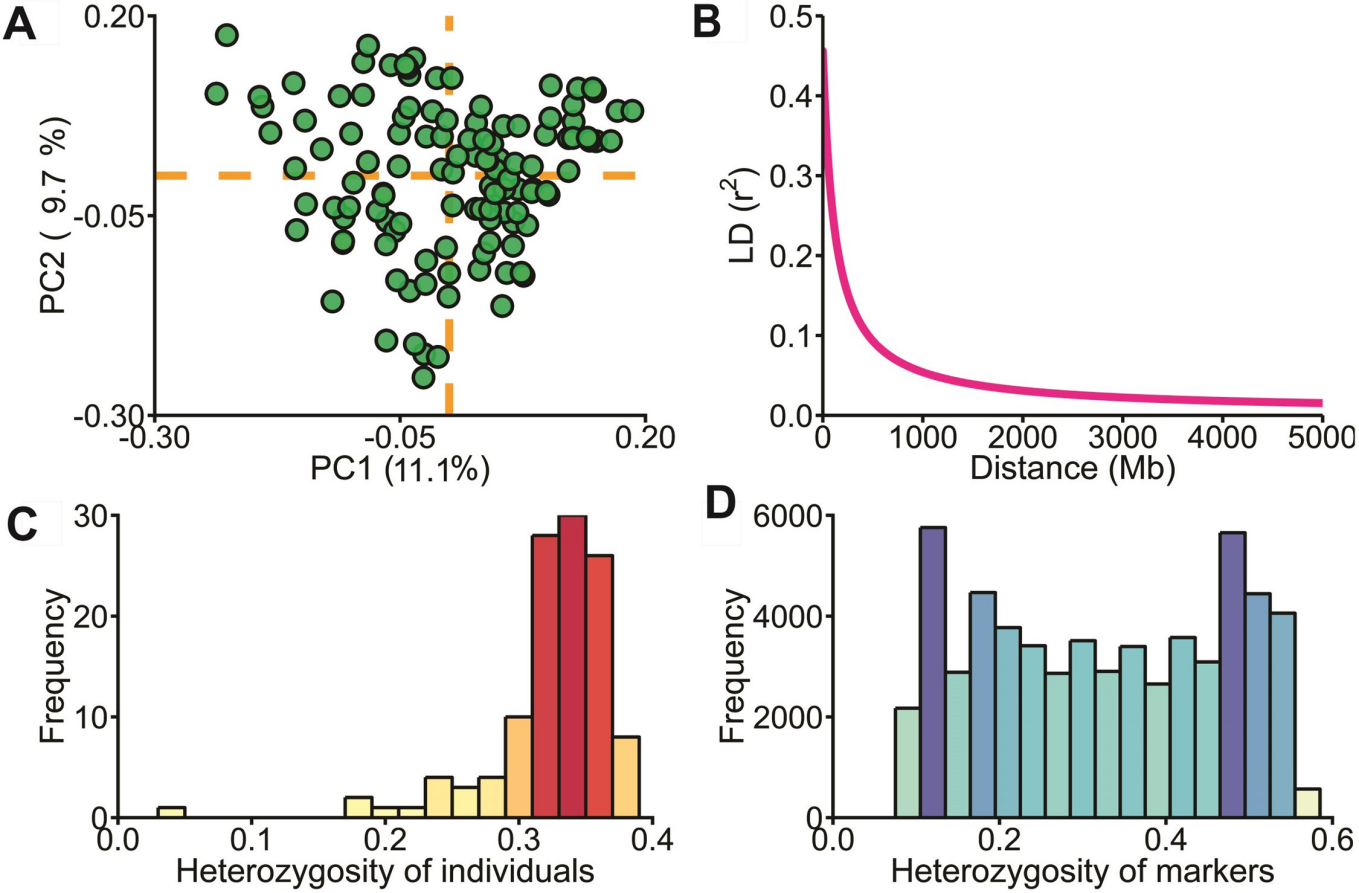
Population structure and genomic data. (A) Population structure of the 118 maize hybrids revealed by the first two principal components of 59,215 SNP markers. (B) LD decay across the whole genome. (C) and (D) heterozygosity of individuals and markers, respectively.

The genetic structure of the hybrid panel was accessed by PCA using 59,215 SNP markers (Fig 2A). The first two PCs captured a small percentage of the total variance (20.8%). In addition, the individuals had a wide distribution throughout the projection space, which indicates a weak structure among the genotypes. Moreover, a rapid decline in LD was observed (Fig 2B), with 121.7 kb extent when *r*^2^ reached 0.23 (half the maximum value). The average heterozygosity of hybrids was 0.32, ranging from 0.03 to 0.38 with most of the individuals presenting around 0.35 (Fig 2C). The low values found for some individuals indicate that some inbred lines used in the diallel crosses were of high genetic similarity. For the heterozygosity of markers, this value was also 0.32, varying from 0.10 to 0.61 (Fig 2D).

### Marker-trait associations

The additive and heterozygous (dis)advantage GWAS models were used to dissect the genetic basis of the traits RDM, RV, RAD, SRL, and SRSA under N stress and N stress plus *A*. *brasilense* conditions, since for these traits the genotypes showed a differential performance due to the inoculation effect. Only the genetic relatedness (K matrix) was used as a covariate in all GWAS analyses.Thus, the population structure information was not included due to the increase in the deviation from the expected *p*-values showed by Q–Q plots (not presented). Furthermore, based on the LD decay for this hybrid panel, the gene annotation was performed within a 50 kb sliding window around each significant SNP.

Concerning the additive GWAS model, eight significant SNP-trait associations were revealed in the maize hybrids evaluated under N stress plus *A*. *brasilense* (Table 1 and S2 and S6A Figs). In general, at least one candidate gene was identified for each trait, which was located on the chromosomes 2, 4, 6, 7, and 9. In addition, using the same model but for N-stress treatment, one significant association was detected for each trait, totaling five candidate genes, which were located on chromosomes 2, 5, and 6 (Table 1 and S3 and S6B Figs). However, for chromosome 5, position 149998432, no candidate gene was found within the window considered. The results for RSR in both treatments were disregarded due to poor adjustment with the expected values shown by the Q–Q plot.

**Table 1:** List of candidate genes around of significant SNPs identified by additive GWAS model.

Trait	Candidate gene	SNP	Chr.	Position	MAF	Effect	*P*-value	Gene annotation
	***N stress plus Azospirillum brasilense***							
RDM	Zm00001d051881	T/C	4	173317340	0.14	0.20	5.50x10^-10^	Protein BTR1
RDM	Zm00001d035859	T/C	6	56793578	0.42	-0.20	1.31x10^-16^	Plastocyanin homolog 1
RV	Zm00001d006108	C/G	2	198321726	0.43	-3.61	1.61x10^-21^	Hydroxyproline-rich glycoprotein family protein
RAD	Zm00001d013098	A/G	5	4668442	0.46	0.04	3.14x10^-24^	Aldehyde oxidase 2
RAD	Zm00001d046604	T/C	9	98488802	0.32	0.02	3.52x10^-11^	(Z)-3-hexen-1-ol acetyltransferase
SRL	Zm00001d005892	A/G	2	191920029	0.48	-386.6	1.40x10^-09^	Ethylene-responsive transcription factor ERF109
SRL	Zm00001d020747	T/C	7	131108804	0.34	-357.7	1.98x10^-08^	Aquaporin TIP4-1
SRSA	Zm00001d052221	A/G	4	183616939	0.29	83.61	4.13x10^-10^	Tetratricopeptide repeat (TPR)-like superfamily protein
	***N stress***							
RDM	Zm00001d013098	A/G	5	4668442	0.46	0.02	3.14x10^-24^	Aldehyde oxidase2
RV	Zm00001d038300	A/T	6	153844954	0.32	-1.97	2.89x10^-13^	Putative cytochrome P450 superfamily protein
RAD	Zm00001d005892	A/G	2	191920029	0.48	0.02	1.40x10^-09^	Ethylene-responsive transcription factor ERF109
SRL	Zm00001d002930	A/G	2	27052534	0.21	487.5	3.24x10^-11^	Hypothetical protein
SRSA	-	A/C	5	149998432	0.40	86.9	2.93x10^-10^	There is no candidate gene in the region

Description of candidate genes found for root dry mass (RDM), root volume (RV), root average diameter (RAD), specific root length (SRL), and specific root surface area (SRSA) evaluated in maize hybrids under N stress and N stress plus *Azospirillum brasilense*. Chr: Chromosome and MAF: Minor allele frequency.

Two candidate genes identified in the inoculated treatment were similar to those identified under N-stress treatment, but for different traits. In this sense, the candidate genes Zm00001d013098 and Zm00001d005892 were related to RAD and SRL under *A*. *brasilense* treatment, and to RDM and RAD under non-inoculated treatment, respectively.

**Fig 3:**
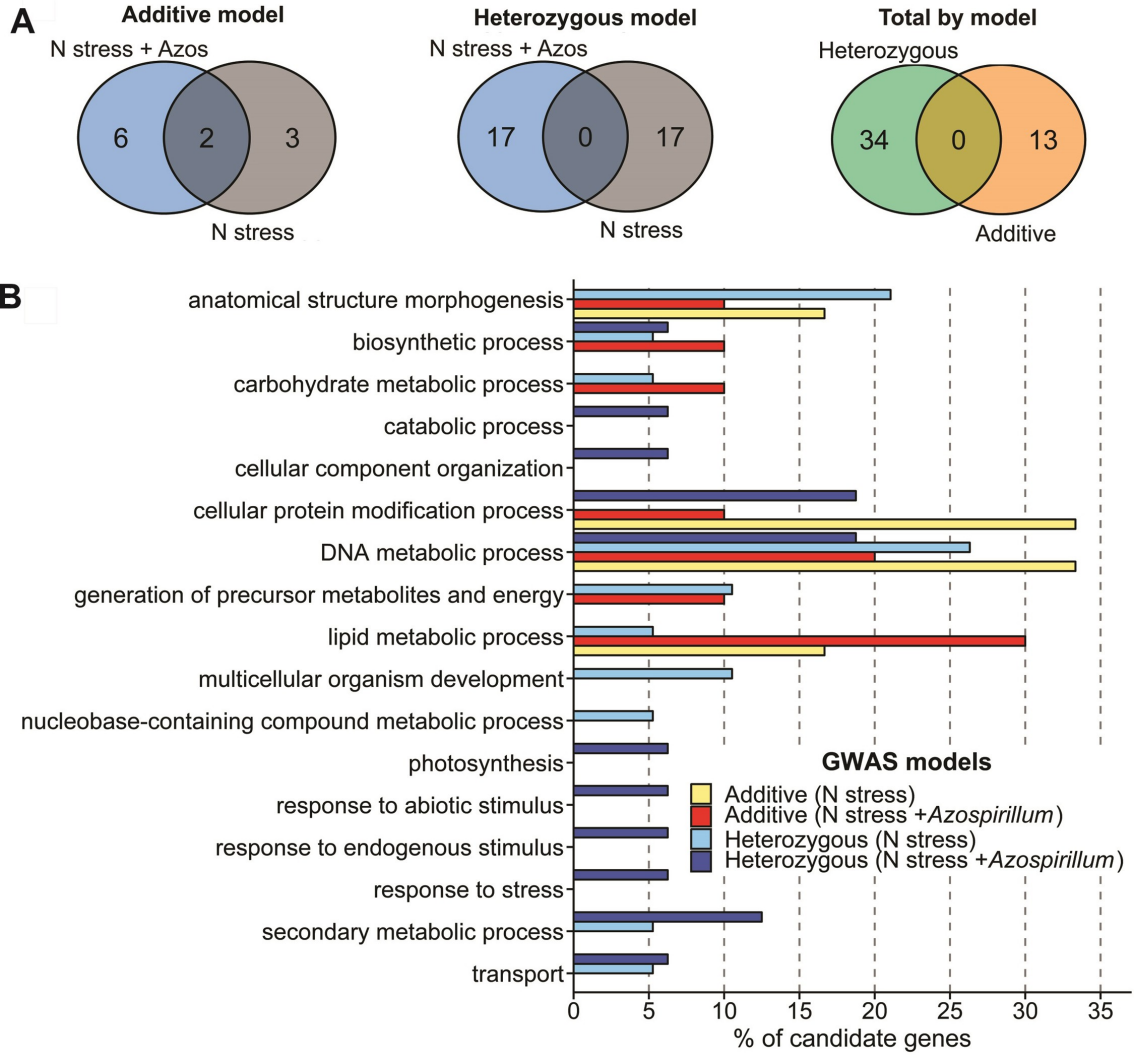
Candidate genes found by different GWAS models for N stress and N stress plus *Azospirillum brasilense*. (A) Venn diagrams showing the unique and shared significant SNPs. (B) Enriched GO terms.

A higher number of significant associations were revealed using heterozygous (dis)advantage GWAS model. Seventeen significant SNPs were found associated with traits under N stress plus *A*. *brasilense* treatment located on chromosomes 1, 2, 3, 7, and 8 (Table 2 and S4 and S7 Figs). Several common candidate genes were found among the traits: Zm00001d029115 (RDM, RV, and RSR), Zm00001d037182 (RDM and RV), Zm00001d003312 (RV and RAD), and Zm00001d030590 (RAD and SRL). Under N stress, 17 significant associations were identified throughout the chromosomes 1, 2, 3, 4, 5, 6, and 9 (Table 2 and S5 and S8 Figs). For this model, any of the candidate genes were detected simultaneously for both inoculated and non-inoculated treatments (Fig 3A). No candidate genes were detected for chromosome 1 position 251090900 (RAD, inoculated treatment) and chromosome 3 position 165642810 (RDM, non-inoculated treatment).

**Table 2:** List of candidate genes around of significant SNPs identified by heterozygous (dis)advantage GWAS model.

Trait	Candidate gene	SNP	Chr	Position	MAF	APHo	APHe	*P*-value	Gene annotation
	***N stress plus Azospirillum brasilense***								
RDM	Zm00001d029115	T/C	1	58456902	0.09	1.34	1.51	1.92x10^-10^	Protein strictosidine synthase-like
RDM	Zm00001d037182	C/G	6	114938556	0.10	1.40	1.26	1.07x10^-11^	12-oxo-phytodienoic acid reductase3
RV	Zm00001d029115	T/C	1	58456902	0.09	17.75	20.31	4.37x10^-13^	Protein strictosidine synthase-like
RV	Zm00001d032763	A/G	1	237658345	0.19	18.50	17.71	1.57x10^-08^	Pre-mRNA-processing factor 19 homolog 2
RV	Zm00001d003312	T/G	2	39796017	0.19	18.70	17.37	5.56x10^-08^	3-ketoacyl-CoA thiolase 2 peroxisomal
RV	Zm00001d037182	C/G	6	114938556	0.10	18.67	16.28	3.58x10^-13^	12-oxo-phytodienoic acid reductase3
RAD	Zm00001d030590	A/G	1	146746338	0.17	0.66	0.69	3.02x10^-13^	Hypothetical protein
RAD	-	T/G	1	251090900	0.22	0.67	0.66	1.57x10^-08^	There is no candidate gene in the region
RAD	Zm00001d003312	T/G	2	39796017	0.19	0.68	0.65	2.79x10^-09^	3-ketoacyl-CoA thiolase 2 peroxisomal
RAD	LOC103636767	T/C	8	14392135	0.18	0.66	9.68	5.99x10^-11^	Formin-like protein 20
SRL	Zm00001d030590	A/G	1	146746338	0.17	4364.2	4079.0	2.25x10^-10^	Hypothetical protein
SRL	Zm00001d002736	T/C	2	20783203	0.18	4179.5	4433.6	2.52x10^-09^	Carotenoid cleavage dioxygenase7
SRL	Zm00001d008828	T/C	8	21875974	0.18	4146.3	4485.5	1.02x10^-11^	Uncharacterized loci
SRSA	Zm00001d033957	A/T	1	27957495	0.20	656.8	680.9	1.86x10^-08^	Helix-loop-helix DNA-binding domain containing protein
RSR	Zm00001d029115	T/C	1	58456902	0.09	0.20	0.26	9.11x10^-12^	Protein strictosidine synthase-like
RSR	Zm00001d043812	A/G	3	210821486	0.20	0.21	0.20	6.28x10^-1^	Isopentenyl transferase3B
RSR	Zm00001d020647	T/C	7	126412420	0.23	0.22	0.19	7.50x10^-08^	Phospholipid:diacylglycerol acyltransferase 1
	***N stress***								
RDM	Zm00001d003331	T/C	2	40341681	0.22	1.41	1.32	9.16x10^-11^	Putative WRKY transcription factor 34
RDM	Zm00001d006036	A/G	2	195919131	0.27	1.40	1.34	3.55x10^-08^	Heat shock 70 kDa protein 9 mitochondrial
RDM	-	T/C	3	165642810	0.21	1.41	1.32	5.96x10^-08^	There is no candidate gene in the region
RDM	Zm00001d044754	A/T	9	1340463	0.17	1.41	1.30	4.52x10^-11^	Pyrophosphate—fructose 6-phosphate 1-phosphotransferase subunit beta 2
RV	LOC109941493	T/C	1	162580315	0.12	17.59	20.17	7.31x10^-16^	Plasma membrane ATPase 2-like
RV	Zm00001d036118	T/C	6	72999857	0.26	17.63	18.74	1.09x10^-09^	Putative homeobox DNA-binding and leucine zipper domain family protein
RAD	Zm00001d006722	T/C	2	215259958	0.23	0.68	0.65	3.50x10^-08^	Arabinosyltransferase RRA3
RAD	Zm00001d037514	T/C	6	127764798	0.21	0.68	0.65	1.49x10^-09^	Putative uncharacterized mitochondrial protein
SRL	LOC100279630	T/C	1	4994455	0.15	4117.5	4646.4	8.56x10^-17^	MADS-box transcription factor family protein
SRL	Zm00001d029134	T/C	1	59906568	0.25	4401.4	4138.5	5.25x10^-09^	CW-type Zinc Finger
SRL	Zm00001d029247	T/C	1	63585160	0.12	4151.8	4632.4	2.23x10^-14^	ARM repeat superfamily protein
SRL	Zm00001d029385	T/C	1	68562928	0.25	4429.5	4115.7	1.74x10^-07^	AAA-type ATPase family protein
SRL	Zm00001d030287	T/C	1	119697532	0.25	4194.0	4343.3	2.67x10^-08^	Protein CLT2 chloroplastic
SRL	Zm00001d013070	T/C	5	4219053	0.25	4300.5	4239.4	7.64x10^-09^	Transcription factor MYB98
SRL	Zm00001d037596	A/T	6	130932567	0.20	4445.7	4013.6	1.78E-07	RING/U-box superfamily protein
SRSA	LOC100279630	T/C	1	4994455	0.15	636.8	739.5	1.99x10^-13^	MADS-box transcription factor family protein
RSR	Zm00001d051886	C/G	4	173630271	0.15	0.18	0.22	1.04x10^-13^	Putative MATE efflux family protein

Description of candidate genes found for root dry mass (RDM), root volume (RV), root average diameter (RAD), specific root length (SRL), and specific root surface area (SRSA) evaluated in maize hybrids under N stress and N stress plus *Azospirillum brasilense*. Chr: Chromosome, MAF: Minor allele frequency, APHo: Average phenotype of individuals with homozygous genotype, and APHe: Average phenotype of individuals with heterozygous genotype.

In total, 47 significant SNP-trait associations were found, where 25 were related to traits under N stress plus *A*. *brasilense* and 22 to N stress. Regarding the models, 13 significant associations were identified by using the additive GWAS model and 34 the heterozygous (dis)advantage model. There was no candidate gene shared between them (Fig 3A). Finally, the nature of the SNP effect on the traits, positive or negative, was independent of the treatment or GWAS model (S6, S7, and S8 Figs).

The categorization of candidate genes sequences according to biological process using the Blast2GO software showed that just one biosynthetic category was present in all treatments (Fig 3B). Moreover, in general, the candidate genes found by additive GWAS model tended to be mainly enriched for terms such as “DNA metabolic process” and “lipid metabolic process”. In turn, those found by the heterozygous (dis)advantage model showed more exclusive biological functions; for example, “catabolic process”, “cellular component organization”, “response to stress”, and “secondary metabolic process”. Comparing the inoculated and non-inoculated treatments, a different pattern of categorization was seen between them, especially for the candidate genes found by the heterozygous (dis)advantage model.

## Discussion

### Genotypic variation of maize to *A*. *brasilense* under nitrogen stress

One of our aims was to evaluate the genetic variability of the responsiveness of maize hybrids to the inoculation with the PGPB *A*. *brasilense* and the genetic control of related traits to this effect. The few studies that have reported the differential responsiveness among maize genotypes to *A*. *brasilense* inoculation were based on a smaller number of hybrids or inbred lines [5,6,54]. Moreover, as far as we know, our report has evaluated the largest number of maize genotypes for their association with PGPB.

In general, *Azospirlillum* spp. promotes several benefits and changes in maize, including phytohormone production of auxins, cytokinins, and gibberellins [55,56], plant growth and yield [35,55], contents of secondary metabolites [57], photosynthetic potential [1], anatomical pattern (e. g. metaxylem vessel elements) and architecture of roots [31,58], N_2_ fixation [6], fertilizer-N recovery [59], tolerance of abiotic stresses (e. g. N limitation and drought conditions) [55,60]. In this work, the inoculation of *A*. *brasilense* under N stress promoted significant change in maize performance for six root-related traits: RDM, RV, RAD, SRL, SRSA, and RSR. Some studies have also shown the positive effect of the inoculation of *Azospirillum* spp. on RDM, RV, and the promotion of thinner root growth [55,61,62], but our study reports for the first time the response in SRL and SRSA.

Our results did not show pronounced differences among the distributions of adjusted means of the hybrids under N stress and N stress plus *A*. *brasilense*. However, we observed a significant variation in the delta (the difference between inoculated and non-inoculated treatments), including some of the maize hybrids showing negative effects on the traits due to the inoculation. This result shows that adding only one PGPB to the microbiome is enough to expand the range of maize plant responses under low-N stress. This may be because microbes alter the plant functioning and confer different characteristics to the host plant, reinforcing the emerging idea of the holobiont being a unit of selection that possess a larger variability to be explored in plant breeding [63–65].

Studies reporting a decrease in the phenotypic traits of host plants due to the inoculation with PGPBs, such as *A*. *brasilense*, are not common in the literature [66]. One possibility is that the genotypes with a negative response to inoculation can have more unfavorable alleles related to the association with *A*. *brasilense*. For example, triggering plant defense responses incurs an energetic cost [67], which may lead to a reduction in resources to root system development, causing a worse growth than only the N-stress condition would already entail. In addition, similarly to the plant–endophyte interactions, the “balanced antagonism theory” applies to the plant–PGPB relationships [68,69]; then, phenotypic plasticity in the host plants may vary from mutualism to antagonism depending on the plant genotype, the environmental conditions, and the bacterial strain.

Another explanation for the negative responsiveness is because the effect of *A*. *brasilense* on the plant can vary according to the concentration of the inoculant [66,70]. In general, plant hormones are stimulatory only at certain concentrations, which should not exceed the stimulatory threshold specific to each plant genotype [71]. The higher concentration of *A*. *brasilense* in the root environment may increase the release of plant hormones that consequently inhibit root growth [66]. Thus, considering the number of genotypes evaluated, the concentration of the inoculant used in our experiment could be unfavorable for some of them, even at the recommended dose.

On the other hand, the reduction in root traits due to inoculation would not necessarily be a negative factor for the plant. Under abiotic stress conditions, such as low N supply and drought, high root/shoot ratios are common [72,73]. In this sense, we found moderate positive correlations between the ΔRDM and ΔRV root traits and ΔRSR, which indicates that under *A*. *brasilense* inoculation some plant genotypes could reduce the investment in root growth in order to allocate it to shoot development. However, further studies are needed to better understand the influence of inoculation with this PGPB on the distribution of dry matter between roots and shoots.

The continuous phenotypic variation and the moderate estimates of heritability for the traits related to the maize responsiveness to *A*. *brasilense* suggest the influence of several genes of small effect and a strong environmental influence. In summary, these results reinforce the complex PGPB × plant × environment interactions. Furthermore, they show the possibility of improving plants to be more efficient by the association with PGPB.

### Candidate genes related to the maize responsiveness to *A*. *brasilense*

To the best of our knowledge, this is the first report employing GWAS to assess the genetic architecture of the association of maize with *A*. *brasilense*. Several candidate genes related to the maize responsiveness to *A*. *brasilense* were detected. Considering the panel size used in our study, possibly the power of our GWAS analyses has been low and only theose SNPs with more greater effect have been identified [74]. Korte & Farlow [75] suggest that a way to mitigate the small sample effect is to account for large phenotypic variability. Thus, as we used hybrids rather than inbred lines, a series of different allelic combinations can occur, increasing the genetic variants with heterozygous loci and thereby allowing the finding of better results in GWAS analysis [76]. This is reflected in the number of significant SNPs identified by the heterozygous (dis)advantage model, which was about three times higher than the additive model. Consequently, given the high number of candidate genes found, we focused our discussion mainly on those with functions more related to the treatments of this study.

It is known that the colonization of host plants by beneficial microbes depends on their ability to manipulate defense-related pathways [4]. In this study, the candidate gene Zm00001d051881 (additive model) was found, which codes the protein Binding to ToMV RNA 1 (BTR1). This is involved in the defense against Tomato Mosaic Virus (TOMV) RNA, with possible indirect effects on the host’s innate immunity [77]. In addition, Zm00001d052221 (additive model) codes the tetratricopeptide repeat (TPR)-like protein superfamily, which is determinant for the transduction of signals mediated by plant hormones and able to activate the plant’s defense response. For example, TPR is related to the quantitative resistance of soybean to *Fusarium graminearum* [78]. Another candidate gene is the ethylene-responsive transcription factor ERF109 (Zm00001d005892, additive model), which besides being involved in ethylene-activated abiotic stress responses [79], induces the expression of defense-related genes promoting a positive modulation of the response against pathogen infections [80]. Gene Zm00001d029115, identified for two traits using the heterozygous (dis)advantage model, codes the protein strictosidine synthase-like, known to play a key role in the alkaloid biosynthesis pathway. These chemical compounds function as protection against pathogenic microorganisms and herbivorous animals. In addition, the improvement in alkaloid content in the roots has been observed with *A*. *brasilense* inoculation in medicinal plants [81], but there are no reports about its induction in cereal crops.

The modulation of plant hormones and related signaling pathways by *A*. *brasilense* are aspects also frequently reported [2,61]. For example, we found Zm00001d013098 (additive model) corresponding to Aldehyde oxidase 2, which is a key enzyme in the final step of abscisic acid (ABA) biosynthesis. In addition, it performs the final catalytic conversion of indole-3-acetaldehyde (IAAld) in indole-3-acetic acid (AIA) in different tryptophan-dependent auxin biosynthesis pathways [82]. Moreover, we found the candidate gene for 12-oxo-phytodienoic acid reductases (Zm00001d037182, heterozygous model), which are key enzymes in the control of jasmonate (JA) biosynthesis in plants such as maize [83] and wheat [83]. Among other functions, this phytohormone orchestrates defense and growth responses [84].

Some studies show modulation of the induction and emission of plant volatiles by plant-associated microorganisms, including PGPBs and Rhizobia [85,86]. In turn, these chemicals have an important role, especially on the induction of resistance in plants against insects and pathogens [87,88]. We found the Zm00001d046604 candidate gene (additive model) corresponding to (Z)-3-hexen-1-ol acetyltransferase. This enzyme is involved in the green leaf volatile biosynthetic process that is derived from the lipoxygenase pathway [89]. In agreement with this finding, *A*. *brasilense* negatively affects the attraction of the pest insect *Diabrotica speciose* to maize by inducing higher emissions of the volatile (E)-β-caryophyllene. Therefore, the validation of this candidate gene in further studies could help to understand better the role of plant defense against pests induced by *A*. *brasilense*.

Regarding the candidate genes related to abiotic stress mitigation, we found Zm00001d020747 (additive model), encoding Aquaporin TIP4-1. Under N deficiency, this plant transporter is up-regulated in Arabidopsis [90] and it is induced by rhizobial and arbuscular mycorrhizal fungi symbiosis [91]. In both cases, its function is related to the N delivery between plant compartments.

We found a candidate gene directly involved in plant root growth encoding hydroxyproline-rich glycoprotein family protein (Zm00001d006108, additive model), a protein family from plant cell walls classified as arabinogalactan-proteins (AGPs), extensins (EXTs), and proline-rich proteins (PRPs). It plays a key role in several processes of plant development such as root elongation and root biomass, especially under stress conditions [92]. Additionally, AGPs are exuded in the rhizosphere and help in communicating with soil microbes, participate in the signaling cascade modulating the plant’s immune system, and are required for root colonization by symbiotic bacteria [93]. Another, LOC103636767 (heterozygous model), corresponds to formin-like protein 20, which is involved in cytoskeleton movement and secondary cell wall formation [94].

The major part of N in the leaf is allocated to the chloroplast proteins, and deficiency in this nutrient leads to a reduction in photosynthetic efficiency [95]. The Zm00001d035859 candidate gene (additive model) found in our study is related to the Plastocyanin homolog 1, a protein involved in the transfer of electrons in the photosystem I. In accordance with this result, the inoculation of the PGPB *Burkholderia* sp. in *Arabidopsis thaliana* leads to modification in the expression of this protein [96]. Moreover, it is involved in the response of maize to N deficiency [97].

Although the candidate genes found for the N-stress treatment were not the main focus of this study, many of them have been previously described due to their direct or indirect relation to plant responses to abiotic stress conditions. The LOC109941493 gene (heterozygous model) encodes the plasma membrane ATPase 2-like, this being an ion pump in the plant cell membrane important for root growth and architecture under different nitrogen regimes [98]. In addition, Zm00001d006722 (heterozygous model) is related to arabinosylation of extensin proteins that contribute to root-cell hair growth, these being specialized in the absorption of nutrients [99]. The Zm00001d013098 and Zm00001d038300 genes (additive model) corresponding to Aldehyde oxidase2 and Ethylene-responsive transcription factor ERF109, respectively, were the only candidate genes shared between both treatments. Their functions described above, related to ABA and AIA biosynthesis and the ethylene-activated signaling pathway, are also frequently reported in N availability and hormone interactions [100,101]. Moreover, this suggests that the regulation and signalization of these hormones in the plant can be involved in the cross talk between *A*. *brasilense* and N stress in maize. Therefore, besides these results indicating that the stress applied in our experiment was effective, they also could be helpful in further studies to understand better the genetic control of root traits under N stress in the early stages of plant development for improving tolerance in maize.

Some of the candidate genes found by the heterozygous (dis)advantage GWAS model were identified for more than one trait; this was not observed using the additive model. For these, the effect on phenotypes was always in the same direction; for example, the candidate gene Zm00001d029115 (protein strictosidine synthase-like) had a negative effect on both RDM and RV. Possibly, this occurred because these pleiotropic candidate genes were only found between RDM, RV, and RAD traits which showed a positive correlation among them.

### Additive and heterozygous (dis)advantage GWAS models

GWAS analyses using non-additive models are common in human and animal studies [102–104]. However, few studies have been reported using plant species [21,22]. In our study, most of the significant SNPs were identified by heterozygous (dis)advantage GWAS analyses and none were detected by the additive model, which demonstrates how important it is to study the non-additive effects on the genetic variability of maize responsiveness to both *A*. *brasilense* and N stress. This was also evident through the results of GO terms, where an increase in exclusive biological functions was verified. This indicates that the PGPB provide the plant with a broader spectrum of internal activities, which may be an advantage for growth in stressful environments, such as N deficiency, with possible consequences in plant evolutionary potential.

Furthermore, our results showed that heterozygous genotypes can have advantages or disadvantages on the root traits (both treatments) depending on the allelic combinations that are formed by the parental crossing. Thus, the strategy of use of SNP‒trait associations found by heterozygous loci in breeding programs depends on the effect of the heterozygous genotype. This is a challenge to plant breeders because during hybrid development the allele combination should be predicted by parental selection in order to benefit its association with PGPB. In this sense, further studies underlying these candidate genes are required to understand better the biological mechanisms of heterotic performance, in comparison to homozygous, in the presence of these PGPB. For those providing an advantage, the alleles should be improved separately in different heterotic groups for their subsequent combination in the mating process. On the other hand, when the heterozygous genotypes are a disadvantage, one or other allele should be improved simultaneously in both heterotic groups in order to obtain homozygous genotypes in hybrids.

## Conclusions

Our study modeling additive and heterozygous (dis)advantage effects in GWAS analyses revealed 25 candidate genes for the responsiveness of maize to *A*. *brasilense*, with key roles particularly in plant defense, hormonal biosynthesis, signaling pathways, and root growth providing insights into their complex genetic architecture. In this context, non-additive effects contribute substantially to the maize phenotypic variation in response to the inoculation and are related to a wider spectrum of biological functions. Together, these findings allow to be started the marker-assisted selection and genome editing in breeding programs for the development of maize hybrids that can take advantage of this association more efficiently. Finally, our results also represent a benchmark in the identification of homologous genes in important related species, such as rice and wheat, besides advancing the understanding of the genetic basis of plant–PGPB interactions.

## Supporting information

S1 Table Phenotypic data analyses. Wald Test for fixed effects and Likelihood Ratio Test for random effects from the joint diallel analysis of 118 maize hybrids evaluated under N stress and N stress plus *Azospirillum brasilense* treatments.
(DOCX)

S1 FigPearson correlations, variance components and heritabilities.(A) Pearson correlation between maize hybrid adjusted means under N stress and N stress plus *A*. *brasilense*. (B) Estimates of variance components (${\hat{\sigma }}_{G}^{2}$: genotypic variance; ${\hat{\sigma }}_{GY}^{2}$: genotype-by-year variance; ${\hat{\sigma }}_{\epsilon }^{2}$: error variance) and broad-sense heritabilities.(TIF)

S2 Fig Manhattan and Q–Q plots of additive GWAS model for N stress plus *Azospirillum brasiliense*. (A) root dry mass, (B) root volume, (C) root average diameter, (D) specific root length, (E) specific root surface area, and (F) root/shoot ratio.
(TIF)

S3 Fig Manhattan and Q–Q plots of additive GWAS model for N stress. (A) root dry mass, (B) root volume, (C) root average diameter, (D) specific root length, (E) specific root surface area, and (F) root/shoot ratio.
(TIF)

S4 Fig Manhattan and Q–Q plots of heterozygous (dis)advantage GWAS model for N stress plus *Azospirillum brasiliense*. (A) root dry mass, (B) root volume, (C) root average diameter, (D) specific root length, (E) specific root surface area, and (F) root/shoot ratio.
(TIF)

S5 Fig Manhattan and Q–Q plots of heterozygous (dis)advantage GWAS model for N stress. (A) root dry mass, (B) root volume, (C) root average diameter, (D) specific root length, (E) specific root surface area, and (F) root/shoot ratio.
(TIF)

S6 Fig Box plot of significant SNPs identified by additive GWAS model. (A) N stress plus *Azospirillum brasiliense* and (B) N stress.
(TIF)

S7 Fig Box plot of significant SNPs identified by heterozygous (dis)advantage GWAS model for N stress plus *Azospirillum brasiliense*. (TIF)

S8 Fig Box plot of significant SNPs identified by heterozygous (dis)advantage GWAS model for N stress. (TIF)
