# Supplementary material for: Additive and heterozygous (dis)advantage GWAS models reveal candidate genes involved in the genotypic variation of maize hybrids to Azospirillum brasilense
Source: PLoS One. 2019 Sep 19;14(9):e0222788. doi: 10.1371/journal.pone.0222788 (PMC6752820; doi:10.1371/journal.pone.0222788)
Supplement: S1 Table — Wald Test for fixed effects and Likelihood Ratio Test for random effects from the joint diallel analysis of 118 maize hybrids evaluated under N stress and N stress plus Azospirillum brasilense treatments. (DOCX) [file pone.0222788.s001.docx]

**S1 Table. Phenotypic data analyses.** Wald test for fixed effects and Likelihood Ratio Test for random effects from the joint analysis of 118 maize hybrids evaluated under N stress and N stress plus *Azospirillum brasilense* treatments.

| Variation source | PH | SDM | RDM | LRL | ARL | RAD | RV | SRL | SRSA | RSR |
| --- | --- | --- | --- | --- | --- | --- | --- | --- | --- | --- |
| *Fixed* |  |  |  |  |  |  |  |  |  |  |
| Year (Y) | 1533.0** | 576.9** | 29.5** | 41.0** | 2.2 | 107.0** | 9.3** | 11.8** | 95.0** | 575.5** |
| Block (B)/Y | 69.0** | 4.0 | 17.9** | 27.8** | 13.2* | 219.0** | 61.4** | 85.8** | 57.3** | 21.7** |
| Countertop/B/Y | 505.0** | 165.6** | 152.4** | 177.9** | 191.1** | 10.0 | 10.4** | 12.4 | 17.2** | 63.3** |
| Inoculation (I) | 0 | 0.1 | 8.3** | 0.2 | 1.1 | 19.0** | 165.4** | 11.8** | 7.0** | 5.1* |
| Y x I | 0 | 0.1 | 0.7 | 1.1 | 0 | 1.0 | 0 | 1.7 | 1.1 | 0.5 |
| *Random* |  |  |  |  |  |  |  |  |  |  |
| Genotype (G) | 3.7 | 0.1 | 16.9** | 7.8** | 49.9** | 37.6** | 39.4** | 28.6** | 23.0** | 115.0** |
| G x Y | 12.1** | 8.8** | 1.5 | 4.5* | 0.8 | 1.3 | 0.5 | 0.7 | 2.8 | 10.7** |
| G x I | 0 | 0 | 0 | 0 | 0 | 0.3 | 0.1 | 0 | 0 | 0 |
| G x Y x I | 0 | 0 | 0 | 0 | 0 | 0 | 0 | 0 | 0 | 0 |

PH: plant height, SDM: shot dry mass, RDM: root dry mass, LRL: lateral root length, ARL: axial root length, RV: root volume, RAD: root average diameter, SRL: specific root length, SRSA: specific root surface area, and RSR: root shoot ratio. Significant at 5% (*) or 1% (**) level.
